# From stigma to increased social acceptance? Living with Machado-Joseph disease in São Miguel, Azores, Portugal

**DOI:** 10.1007/s12687-024-00731-w

**Published:** 2024-08-31

**Authors:** Daniela Couto, Jorge Sequeiros, Manuela Lima, Liliana Sousa, Álvaro Mendes

**Affiliations:** 1 Department of Education and Psychology, CINTESIS@RISE, University of Aveiro, Aveiro, Portugal; 2 CGPP – Centre for Predictive and Preventive Genetics, IBMC – Institute for Molecular and Cell Biology, University of Porto, Porto, Portugal; 3 i3S – Instituto de Investigação e Inovação em Saúde, University of Porto, Porto, Portugal; 4 ICBAS – School of Medicine and Biomedical Sciences, UMIB – Unit for Multidisciplinary Research in Biomedicine, University of Porto, Porto, Portugal; 5 Faculty of Sciences and Technology, University of the Azores, Azores, Portugal

**Keywords:** Ataxia, Discrimination, Genetic disease, Genetic risk, Patient association, SCA3

## Abstract

This study describes the experiences with the stigma attached to Machado-Joseph disease (MJD) in São Miguel Island, the Azores (Portugal). We draw on semi-structured interviews with persons with MJD, family members, healthcare professionals, and direct care providers recruited through the local patient’s association (*n* = 28). Qualitative thematic analysis revealed three main themes: (i) the intense stigma associated with MJD in the past; (ii) the current tendency towards increased openness; and (iii) increased availability of information about MJD and support. The findings suggest that stigmatization was more frequent and intense in the past. Still, there is currently a decrease in the intensity of perceived stigma, accompanied by an increasing awareness about MJD within the community. The local patient’s association is noted for playing a pivotal role in raising awareness about MJD in the community and fostering the confidence of individuals with MJD and their families to engage socially, which may help to reduce or mitigate feelings of stigma. This raises questions about whether the diminished stigma towards MJD in São Miguel results from heightened awareness about the condition, a decrease in the social acceptability of stigma, or a gradual internalization and normalization of stigma among individuals with MJD as a coping mechanism.

## Introduction

Machado Joseph disease (MJD) (also known as spinocerebellar ataxia type 3, SCA3) is a dominantly inherited, multi-system late-onset neurological disease (Coutinho and Andrade 1978; Coutinho 1992), which remains without treatment (Matos et al. 2019). MJD was first described in the 1970s, in three families of Azorean ancestry (Machado, Thomas, and Joseph) living in the United States (Nakano et al. 1972; Rosenberg et al. 1976; Woods and Schaumburg 1972) The clinical heterogeneity in these families led to the initial description of three apparently independent diseases. The subsequent identification by Paula Coutinho and Corino Andrade of several large Portuguese kindreds from the Azorean islands of Flores and São Miguel, in Portugal, showed that the three forms of the disease could be found in the same family (Coutinho and Andrade 1978). This paved the way for the unification of the disease, called Machado-Joseph (Coutinho and Sequeiros 1981), the surnames of the first and the largest families described (the Thomas family never wanted their name associated to the disease). MJD was therefore considered to be a single genetic entity with phenotypic variability (Coutinho and Sequeiros 1981; Sequeiros 1989; Coutinho 1992). It is noteworthy that MJD was known in São Miguel as “Ponta Ruiva’s disease”; Ponta Ruiva, in Flores, eventually became an emptied village (Sequeiros 1989).

MJD’s main symptoms include progressive motor impairment, incoordination of gait, speech, swallowing and fine movements of the hands, muscle atrophies, and limitation of eye movements (progressive external ophthalmoplegia); as well as dystonic posturing, bulging eyes, face and tongue fasciculations, and other minor signs. MJD gradually leads to a complete loss of autonomy in daily living (Bettencourt and Lima 2011; Coutinho and Andrade 1978; Lima and Coutinho 1980; Coutinho 1992). Confirmed carriers in the Azores showed an average age at onset of 36.5 (± 9.55 SD) years (range: 16 to 60 years of age) (Lima et al. 2023). MJD is the most frequent spinocerebellar ataxia worldwide, with higher frequencies described in Brazil, Portugal, and China (Sequeiros et al. 2012). MJD prevalence in mainland Portugal is 3.1:100,000 (Coutinho et al. 2013). In the Azores, the prevalence is 39:100,000, with the highest prevalence worldwide reaching 633:100,00 in the island of Flores (de Araújo et al. 2016). Two main intragenic haplotypes (Flores or Joseph, and Machado or São Miguel) show two different ancestral origins (Gaspar et al. 2001); the Joseph haplotype is found in most MJD families worldwide, while the Machado haplotype is mainly seen in families originating from São Miguel or parts of mainland Portugal (Martins and Sequeiros 2018; Martins et al. 2007).

Research has showed that stigma has historically discredited individuals with MJD and their families. Boutté (1987) conducted anthropological research in the island of Flores in the Azores and among Portuguese-Azorean communities in the US. She described how incest and syphilis were attributed as causes of the condition, contributing to a social perception of moral flaws associated to MJD families. Families living with MJD, especially individuals with manifest symptoms, often lived excluded from social life under the shadow of a stigmatizing community (Boutté 1987). Boutté also noted that non-afflicted families on the island were apprehensive about the introduction of the MJD-altered gene into their hereditary pool, leading them to discourage romantic relationships between their members and individuals at risk of the condition (Boutté 1990). Studies on the psychological impact of pre-symptomatic testing (PST) for MJD in the Azores suggest that knowing the test result could help individuals cope with or avoid stigma. Gonzalez and colleagues (2004; 2012) hypothesized that residents of Flores underwent PST to seek proof of their non-carrier status, thereby affirming their social integration within the community. The authors suggested that the patterns of stigma may be different between Flores and São Miguel due to cultural factors specific to these islands; in São Miguel, stigma is thought to be mainly intrafamilial, with families typically refraining from speaking about MJD outside their immediate family circle (Gonzalez et al. 2012). Increasing social exclusion and isolation are described as contributing to excessive alcohol consumption among affected individuals in São Miguel. This, in turn, reinforced perceptions of inadequacy within the community and intensified social exclusion (Soares 2016). Similarly, a study conducted in mainland Portugal described that individuals showing initial symptoms of MJD were often labelled as “drunk” by the community, which led to interpersonal difficulties and problems in employment (Paúl et al. 1999).

Direct mutation analysis for MJD has been available since 1995; a national program of PST for MJD was established in Portugal in 1996 (and later extended to all other late-onset neurological diseases), offering genetic counseling and psychosocial support to at-risk individuals and their families (Maciel et al. 2001; Sequeiros 1996). The local patient support group Associação Atlântica de Apoio aos Doentes de Machado-Joseph (AAADMJ) was created in São Miguel in 1996 and remains as the only specialized community facility in Portugal dedicated specifically to MJD families (Couto et al. 2022). Currently, there is considerable attention from the MJD community to the prospects of drug development and to the clinical trials that are underway (Duarte-Silva et al. 2024). Hope in future scientific advancements has been described as an important resource that fosters resilience and psychosocial adaptation (Mendes et al. 2021). While studies on the psychological adaptation of MJD pre-symptomatic carriers have consistently reported stigmatization as a significant concern (Gonzalez et al. 2004, 2012), there are no published studies specifically addressing experiences with stigma related to living with MJD in São Miguel. This study aimed to contribute to fill in this gap by exploring the perspectives of living with MJD of individuals with a diagnosis and their family members, as well as healthcare professionals (HCP) and direct care providers (DCP).

## Materials and methods

This qualitative exploratory study draws from a larger study examining experiences with MJD in São Miguel, Azores, Portugal. We present the sub-corpus of data focusing on the experiences within the community, a relevant theme observed during that analysis. The study has obtained ethics approval.

### Recruitment

Recruitment was mediated by the AAADMJ. We presented the study in detail to the association’s representatives, who then circulated the information among users, DCPs, and collaborating HCPs. The inclusion criteria were adult individuals, living in São Miguel, with (personal, familial, or professional) experience with MJD, and able to consent. The director of the AAADMJ mediated the contact between those wishing to participate and the researcher (DC) by scheduling a face-to-face or phone meeting. In this first contact, the researcher explained the study in detail and the collaboration requested to potential participants. Informed consent was then obtained from interested participants, and interviews were scheduled at the participants’ convenience. Snowball sampling complemented recruitment.

### Participants

This study comprised 28 participants (18 women): 15 persons with a diagnosis, 5 family members (two non-biological family members, two pre-symptomatic carriers, and one at-risk), 5 DCP, and 3 HCP. The participants’ mean age was 43.8 years (range: 20–70 years). The level of education ranged from 4 years of schooling to university degree. Twenty-one participants were married/in a relationship, and 5 were single; twenty-four participants had children, and 9 had grandchildren. HCP and DCP had 5 to 28 years of experience working with MJD. To preserve participant anonymity, we have limited the description of social and demographic information.

### Data collection

Semi-structured interviews were conducted face-to-face at AAADMJ facilities or by phone by DC from January to March 2022. All interviews fully complied with the sanitary measures required by the Regional Secretary of Health for COVID-19. All interviews were audiotaped with participants’ authorization (range: 6–43 min) and fully transcribed in European Portuguese. Eighteen interviews had a duration of 6 to 15 min. Most participants had dysarthria and showed difficulties in articulating their speech; in these cases, interviews were not extended to avoid impacting further on participants’ well-being. There were also some family members who stated they did not wish to explore some of the interview topics deeper, to which the interviewer complied.

First, social and demographic information was collected (age, sex, education, marital status, number of children and grandchildren), disease status (person with a diagnosis, pre-symptomatic carrier, non-carrier, at 50% risk, non-biological family member), and the relation with MJD (patient, family member, HCP, or DCP); HCP and DCP were asked about the years of experience in the field. Next, an open question invited participants to share their experiences of living with MJD in the community. The interviewer was flexible in exploring the issues that emerged in participants’ accounts.

### Data analysis

Data were submitted to inductive thematic analysis (Braun and Clarke 2006). This method allows for examining the perspectives of different participants, highlighting similarities and differences (Braun and Clarke 2006). First, DC read and re-read all the interview transcripts (in Portuguese) and started the initial open coding by exploring the content in relation to the study objective. Next, DC shared the open coding with LS and ÁM, and the three researchers proceeded to break the data down to identify the most relevant information and themes. The three researchers then met to discuss the themes and to search for connections and patterns between them, considering the adequacy of the themes in relation to the objectives of the study. This process of successive refinement ended with the selection of the most representative quotes of each theme, which were then translated into English.

## Results

We have identified three main themes: (i) intense stigma associated to MJD in the past; (ii) current tendency towards increased openness; and ii) increased availability of information about MJD and support. Each theme is presented, along with data extracts to illustrate key points. The following codes are used: P, person with a diagnosis; FM, family member (with the additional specification code, PSC for pre-symptomatic carriers, NB for non-biological family member); HCP, healthcare professional; DCP, direct care providers.

### Intense stigma associated to MJD in the past

All participants reported experiences in the community where persons with MJD were publicly labeled as *drunk*. This was attributed to individuals showing the incoordination of movements and imbalance typical of early MJD symptoms: “*When patients start to show the first symptoms*,* it’s relatively common that they hear comments like ‘oh*,* s/he had so much drinking already’ or ‘s/he is so drunk!’* [HCP1]. These comments are felt by the individuals with a diagnosis and their family members as offensive and judgmental, making them feel sad and diminished: *“When I heard comments like ‘she looks drunk’ I just wanted to get home and cry”* [P2]; “*People commented I always walk around drunk. I get sad because they don’t know what’s going on”* [P12]. Participants attributed these offensive comments to a lack of knowledge about MJD. However, they also sensed that these comments are decreasing as more people in the community become aware of MJD and its symptoms. Some participants described other attitudes that negatively impact people with MJD and their families. The most common of these attitudes is when participants sense other people staring and/or making comments that suggest pity, which makes them feel different and offended: *“Sometimes they don’t say anything offensive really*,* it’s just the way they stare”* [P3]; *“What hurts me the most is when you hear people saying ‘oh*,* poor thing’. I just hate that*,* no matter who it is!”* [HCP2]. Another comment which is felt negatively is when someone says that the person with MJD will “get better”; these comments are often not interpreted as supportive but instead as revealing a lack of understanding about MJD being progressive and (currently) incurable, which leads participants feeling misunderstood and isolated:

“They say things as ‘have faith in God and trust that he’ll make you getting better’ and I say it’s not like that. I know the disease doesn’t get any better, it only gets worse. We don’t like to hear this.” [P2].

“There are still people who have no idea of what this disease is. When they ask ‘is your husband getting any better?’ I just say ‘I’m sorry but my husband simply will not get any better, this disease will only get worse’. They simply don’t know what this disease is about.” [FM-NB1].

Some participants commented on the decision of people at-risk or pre-symptomatic carriers to have biological children without taking any action to prevent genetic risk in offspring. A few participants disapproved of that decision: “*There are these young girls who are carriers and are getting pregnant. Then they’ll have to take care of the baby knowing in the future they are going to need help”* [DCP7]. Other participants, although questioning those reproductive options, showed more acceptance that *“everyone has the right to live life in their own way and choose to have children if they wish so no matter what”* [DCP6].

### Current tendency towards increased openness

Individuals affected with MJD and their families often faced the disease “behind closed doors”, i.e. staying at home. This was described as more frequent in the past but still occurring, being perceived both as a harmful consequence and as a form of protection against being shunned in the community:

“I didn’t leave my home for 7 years because I was so ashamed.” [P16].

“The old generation would simply close themselves at home. They were ashamed and avoiding going out because of other persons’ comments. There is a new generation who keeps with the same mentality. When they start to need a wheelchair they feel a lot of shame also about what others will say. For example, having to be fed in front of other people.”[DCP7].

Nevertheless, the progression of the disease can lead to increased social isolation and withdrawal, exacerbating feelings of closedness. Symptoms such as dysphagia, motor incoordination, and imbalance often make it hard for people to move around and get out of their homes autonomously. Several participants mentioned that, in the last years, the AAADMJ has been pivotal in stimulating that people with MJD feel more confident in getting out and participating in social activities: *“The psychologist and the social worker came to my house and convinced me to get out more often”* [P7]. The association is well known among several families with MJD on the island, which helps to instill a sense of support within the MJD community. When someone receives a PST carrier result or starts experiencing symptoms, they know they can be supported: *“I had three older siblings who had also been here* [AAADMJ]. *It was through them that the psychologist and the social worker knew that I was at home”* [P7]. In addition, the outdoor activities that are promoted by the AAADMJ’s community day-care center help to raise awareness in the community about MJD and to advocate for more social integration and acceptance:

“He [person with MJD] walked into the cafe with me and he was unbalanced. And there was a gentleman starring who said something like ‘so early in the morning and already this drunk’ (…) I didn’t blame the person for not knowing what it was but I felt like I should take the opportunity to let people know the right information. So I just told him ‘that young man unfortunately it’s not drunk, I wish he was. He has Machado’s disease, whose symptoms are this this and that; and unfortunately he’s going to end up in a wheelchair and bedridden’. The man, then joined us at the table and apologized” [DCP9].

### Increased availability of information about MJD and support

Participants noticed that the community in São Miguel has become more informed and supportive. This shift is described as an interactive development: in the past, individuals with MJD often isolated themselves at home to avoid potentially negative social interactions. As a result, this seclusion kept MJD hidden from the broader community, which in turn limited and acceptance of the condition. The increased availability of information about MJD and the support within the community are linked to the work of the AAADMJ. Younger generations are now significantly more informed about research and medical information, primarily through social media.

“Things are now much more open (…) younger generations have more access to new technologies and there is more knowledge about the disease.”[DCP6].

“Currently I think there’s a lot more information. And when there is more information there is more acceptance. You understand things better.” [HCP4].

However, some participants from MJD families feel that information about the condition has not yet been broadly disseminated within the community: *“People around me seem to have no idea of what this disease is about. When I talk to them about the test* [PST] *they often ask questions like*,* ‘what is this*,* what do you have*,* are you going to die tomorrow?’* [FM-PSC2]. It was evident among those from MJD families that some individuals with MJD still feel reluctant to leave home to avoid displaying symptoms publicly: “*People take refuge at home! They hide! And we have no idea how many people on the island have this disease”* [FM-PSC11].

The role of the AAADMJ is crucial in providing support, including housing adaptations to improve mobility and access to medical, psychological, and social care. As one participant noted, *“Whenever I have doubts or problems, I feel a lot of support here* [AAADMJ]. *I talk a lot with them*,* and this is where I’ve been seeking help”* [P10]. The community day-care center offers persons with MJD the opportunity to participate in leisure activities (including some outdoors) and access medical, rehabilitation and psychosocial interventions. This support is also valuable to families, as it provides assistance to family members and offers them some relief from the demanding caregiving responsibilities:

“I enjoy spending all day here. They are all my friends. For me when I come here [day-care center], I feel happier. At least here I feel more distracted and not just thinking in all the things I can’t no longer do.” [P2].

“The association’s provided some relief to families because those who have the disease go through a lot of suffering but those who provide the caregiving suffer too.” [DCP7].

Some participants reported receiving both emotional and practical support from friends, indicating a greater level of openness:

“Many of my friends, after learning that my mother has the disease and that I will have it too, have said ‘hey, that’s tough but we’ll be here for you.’ I have very close friends, true friendships. When you share something difficult, I feel that a true friend will be there for you.” [FM-PSC4].

## Discussion

This is the first study to describe experiences with stigmatization related to MJD in São Miguel, the Azores, from the perspective of individuals with the condition and family members, as well as DCP and HCP. The findings suggest that stigmatization was more frequent and intense in the past. However, there is currently a decrease in the intensity of perceived stigma, accompanied by a growing awareness about MJD within the community. The AAADMJ is described as a key factor in this ongoing process, enabling them to engage more actively in their communities.

Our study suggests that stigmatization of individuals with MJD was perceived as more prevalent and intense in the past, whereas currently there is a greater openness and acceptance from the community. However, *less* perceived or felt stigma does not equate to the absence of stigma. Participants consistently reported observing instances where persons with symptoms being referred to as drunkards, which was seen as offensive and judgmental. Previous studies have reported a similar association between typical symptoms of early MJD (e.g. motor incoordination and imbalance) and attributions to alcohol consumption, both in the Azores (Boutté 1987; Soares 2016) and in mainland clusters, such as the Tagus Valley (Paúl et al. 1999). Similar findings have also been reported for other neurodegenerative diseases involving motor impairment, such as hereditary transthyretin amyloidosis (hATTR) (Oliveira et al. 2017). The disapproving association of MJD with sexual behavior, as described by Boutté (1987) on a different island of the Azores archipelago, was not reported in our study. However, other forms of perceived stigmatization were described, including overt condescending pity. Such experiences have been also reported by individuals with hATTR (Mendes et al. 2017), and people with disabilities (Wang et al. 2015). However, this attitude may indicate increased access to knowledge about MJD, even if that knowledge is sometimes inaccurate. Condescending and patronizing can often be attempts at empathy, or the best reaction individuals can offer without realizing the negative impact it may have on those with the condition (Wang et al. 2015).

This shift towards less perceived stigma is significantly impacting how families with MJD cope with the disease. In the past, families with MJD often lived hidden at home, with individuals showing symptoms withdrawing entirely from community life. Currently, many of these individuals feel less ashamed to appear in public because they perceive the community to be more accepting and aware of MJD. Greater access to information about the condition has contributed to reducing feelings of social stigma.

The AAADMJ is perceived as playing a pivotal role in raising awareness about MJD in the community and advocating for the social integration of MJD families. The association disseminates reliable information about MJD by organizing conferences, participating in studies, providing updates about scientific research, and facilitating informal education activities directly within the community. Additionally, the AAADMJ reaches out to individuals who are still struggling to feel accepted socially while providing psychosocial support. Participants in our study described strategies that help build a trusting relationship between the AAADMJ and families, based on empathy and reciprocity (Sousa and Rodrigues 2012; Sousa et al. 2007).

This study has limitations. First, the small number of participants limits the range of experiences explored. A larger number of participants would allow for the investigation of a broader array of experiences and facilitate comparisons among different individuals involved, thereby enhancing understanding of the disease journey. Second, the recruitment process was facilitated by the AAADMJ, which may have influenced participants’ accounts of their experiences.

In conclusion, our findings suggest that individuals living with MJD in São Miguel experience varying degrees of stigma, with past stigmatization being more intense. The support provided by the AAADMJ is crucial in alleviating the adverse effects of stigmatization on individuals and their families. Receiving support from the association may foster a greater sense of acceptance within the community and reduce feelings of stigma. This raises questions about whether the diminished stigma towards MJD in São Miguel results from increased awareness about the condition, a reduction in the social acceptability of stigma, or a gradual internalization and normalization of stigma among individuals with MJD as a coping mechanism. Future research should focus on exploring the perspectives of individuals from MJD families who do not receive support from the AAADMJ. Additionally, expanding studies to other islands, especially Flores, which has the highest prevalence of MJD worldwide, would offer valuable insights into the experiences of individuals with MJD across different contexts. Those broader scope could help developing more comprehensive strategies to reduce and prevent stigmatization. Engaging more MJD families from São Miguel and other Azorean islands could be greatly assisted by patient support organizations such as the AAADMJ and the Portuguese Association for Hereditary Ataxias.

## Data Availability

Data is provided within the manuscript. The authors do not have permission to share data.
